# Phenomics as an approach to Comparative Developmental Physiology

**DOI:** 10.3389/fphys.2023.1229500

**Published:** 2023-08-14

**Authors:** Jamie C. S. McCoy, John I. Spicer, Ziad Ibbini, Oliver Tills

**Affiliations:** School of Biological and Marine Sciences, University of Plymouth, Plymouth, United Kingdom

**Keywords:** phenomics, development, bioimaging, embryonic development, Comparative Developmental Physiology

## Abstract

The dynamic nature of developing organisms and how they function presents both opportunity and challenge to researchers, with significant advances in understanding possible by adopting innovative approaches to their empirical study. The information content of the phenotype during organismal development is arguably greater than at any other life stage, incorporating change at a broad range of temporal, spatial and functional scales and is of broad relevance to a plethora of research questions. Yet, effectively measuring organismal development, and the ontogeny of physiological regulations and functions, and their responses to the environment, remains a significant challenge. “Phenomics”, a global approach to the acquisition of phenotypic data at the scale of the whole organism, is uniquely suited as an approach. In this perspective, we explore the synergies between phenomics and Comparative Developmental Physiology (CDP), a discipline of increasing relevance to understanding sensitivity to drivers of global change. We then identify how organismal development itself provides an excellent model for pushing the boundaries of phenomics, given its inherent complexity, comparably smaller size, relative to adult stages, and the applicability of embryonic development to a broad suite of research questions using a diversity of species. Collection, analysis and interpretation of whole organismal phenotypic data are the largest obstacle to capitalising on phenomics for advancing our understanding of biological systems. We suggest that phenomics within the context of developing organismal form and function could provide an effective scaffold for addressing grand challenges in CDP and phenomics.

## Introduction

It is crucial that we make every effort to identify the grand challenges, the biggest questions in biology. Questions around the development of organismal form and function, and its response to biotic and abiotic factors, presents both opportunity and challenge to researchers, with significant advances in understanding possible through the adoption of innovative approaches to its empirical study. Indeed, our ability to tackle these questions is increasingly reliant on developing technologies for measuring properties of biological systems in new ways (Warburton et al., 2006). The term ‘omics’ is now established in biology as a global approach to measuring biological systems at different levels of organisation, from genomics, transcriptomics and metabolomics at biochemical levels, through to phenomics - measurement of the phenotype at the level of the whole organism (Houle et al., 2010). ‘Omics’ approaches increase the scale, resolution, or breadth of measurements, often providing a so-called ‘global’ measure of biological response at a particular level of organisation. Omics have become one of the central facilitators of addressing grand challenges in biological research.

The use of omics at the subcellular level is now commonplace in every area of biological research. Adoption of biochemical omics has been fuelled by technological innovation, enabling transferrable and scalable approaches that are broadly accessible. However, despite being recognized as a significant opportunity for advancing biological research in the 21st century (Houle et al., 2010; Küultz et al., 2013), phenomics remains comparatively underutilized outside of research areas focused on human health, such as crop sciences and medicine. The reasons for this hiatus are difficult to pinpoint, but there is a renewed sense that phenomics is an approach whose time has come. Consequently, here we explore the synergies between phenomics and animal development generally, with a particular emphasis on developmental physiology, particularly Comparative Developmental Physiology, or CDP (Warburton and Burggren, 2005; Warburton et al., 2006). We then suggest ways in which taking a phenomics approach to development is beneficial not only for addressing grand challenges in CDP and development generally, but also in the development of phenomics itself as an approach to studying complex biological systems.

### What is phenomics?

The phenotype is the ultimate expression of biological organisation at the organismal level. It is also the most visible, striking in its breadth and complexity. Approaches to studying whole-organismal biology typically become operational by restricting what can otherwise be overwhelming complexity, to a relatively small number of observable and tractable traits. Indeed, without such selection, any experimental design rapidly becomes unmanageable. However, the process of deciding what to measure is a key step in experimental design and one that has significant implications for the results and interpretation of any experiment. While we trust that our subject knowledge and biological intuition inform this process, there is undoubtedly a significant element of chance associated with this step of experimental design and one that can affect not only the results of an experiment, but even the trajectory of a researcher’s career (Houle et al., 2010; Lürig et al., 2021).

Here, we define phenomics as “the acquisition of high-dimensional phenotypic data on an organism-wide scale” (*sensu* Houle et al., 2010) and the phenome as “the phenotype of the organism as a whole, including the sum of its morphology, physiology and behaviour” (*sensu* Keller et al., 1992). The term ‘phenome” was first used by Davis (1949) to describe “the phenotype as a whole”. Along with Werner K. Maas, they coined the term to describe, “the sum of extragenic, non-autoreproductive portions of the cell, whether cytoplasmic or nuclear”. This view was advanced by Soulé (1964) who referred to the phenome as ‘the phenotypic analog of the genome’. Despite these earlier uses of the term, more recent studies frequently assign coinage of the term ‘phenomics’ in reference to the study of the phenome, to Steven A. Garan in 1996 (Yu and Fang, 2009; Shi et al., 2014; Jin, 2021). Irrespective of its origins, phenomics is currently contextualised as an approach involving the acquisition of phenotypic information at genome wide scales, and it has frequently been regarded as such in reviews and textbooks (Bilder et al., 2009; Furbank 2009; Houle et al., 2010; Hancock, 2014; Tardeiu et al., 2017).

While the notion of phenomics has been around for more than half a century, its study and use as an approach to biological research has only recently gained significant traction. Houle et al. (2010) highlighted its advantages over traditional approaches to studying the phenotype, and others (e.g., Schork, 1997; Freimer and Sabatti, 2003; Bilder et al., 2009) have described it as the natural complement to genomics. Despite this, much like the Human Genome Project, which faced considerable opposition in the face of the counter-claim that reductionist approaches to the study of selected regions of the genome were sufficient for its understanding, phenomics initially met considerable indifference, perhaps suggesting that current approaches to organismal phenotyping were considered sufficient for addressing biological questions of interest (Houle et al., 2010). It is worth noting that while the Human Genome Project fuelled major advancements in biological understanding and acted as the catalyst for advances in modern medicine (Gibbs, 2020), it also met with opposition and some scepticism (Houle et al., 2010; Moraes and Góes, 2016). However, subsequently, molecular omics has necessitated both significant investment and a change in thinking, moving from a reductionist approach to large integrated analyses of complex biological responses, and it has been associated with step-changes in the bioinformatics used for the acquisition and analysis of these data.

Whilst initial traction was lacking in the progression of phenomics as an approach, technological advancements enabling the acquisition of phenotypic data at the whole organism level has accelerated its integration into multiple streams of biological research. Houle et al. (2010) highlighted that limiting the number of phenotypic traits measured to those with some pre-established functional significance could obscure the identification of potentially important traits implicit in the biological response or endpoint of interest, and that high-dimensional phenotyping was necessary to identify the traits, or combinations of traits, that really matter. Now, phenomics is regularly utilised in characterising the genetic basis of complex traits, for tackling disease (Denny et al., 2010; Pendergrass et al., 2011; 2013; Hebbring, 2014; Özdemir, 2020), selective breeding (Crossa et al., 2021) and in the characterisation of responses to toxicants (Audira et al., 2020; 2021; Hussain et al., 2020). Phenomics is also becoming increasingly utilised in the assessment of responses to environmental change, particularly within the crop sciences (e.g., Warringer et al., 2003; Schnaubelt et al., 2013; Singh et al., 2018; Adhikari et al., 2019; Li et al., 2019; Marsh et al., 2021; Tills et al., 2021; 2022).

The explosion of literature adopting high-dimensional phenotyping approaches in the crop and medical literature in the last decade was facilitated by the development of transferable technologies for the acquisition of phenotypic data at whole organism scales in these systems (Bilder et al., 2009; Finkel, 2009; Furbank and Tester, 2011; Groβkinsky et al., 2015; Tardieu et al., 2017; Grapov et al., 2018; Roitsch et al., 2019; Nabwire et al., 2021). This includes advancements in phenotyping technologies for common model species including *Arabidopsis* (Furbank and Tester, 2011; Vanhaeren et al., 2015), the zebrafish *Danio rerio* (Xu et al., 2010; Pelkowski et al., 2011; Peravali et al., 2018; Spomer et al., 2012), the nematode *Caenorhabditis elegans* (White et al., 2010; Olmedo et al., 2015) and the fruit fly *Drosophila melanogaster* (Dagani et al., 2007; Chung et al., 2010; Levario et al., 2016). In a practical sense, phenomics typically takes the form of sensors, combined with some degree of automation, whether analytical such as computer vision pipelines (Tills et al., 2018; Choudhury et al., 2019), or physical such as robotics for processing samples (Yang et al., 2020). Consequently, adding dimensionality to the measurement of the phenotype may include greater temporal or spatial resolution, thereby increasing the scale of experiments, or integrating a wider range of sensing modalities and analytical approaches. The solution provided by phenomics is largely driven by the interaction between the biological system and experimental design, and owing to the incredible diversity in what we term the “phenotype”, what phenomics “looks like” can vary considerably. For example, within the crop sciences, phenomics can be executed by robotic systems in greenhouses, and automated tractors and drones out in the field (Tardieu et al., 2017; Zhao et al., 2019; Yang et al., 2020). In both environments, the integration of a wide array of high-throughput sensing modalities is often undertaken, and the resulting data has greater dimensionality than would otherwise be possible.

More recently, phenomics has seen application to address questions relating to animal ecology and evolution, much of which has been facilitated by the development and application of computer vision approaches (reviewed by Lürig et al., 2021). Lürig et al. (2021) provide a review of computer vision approaches and their utility in integrating phenomics into the fields of ecological and environmental research, as well as the general workflows associated with this. Application of computer vision-based approaches alleviate many of the bottlenecks associated with the acquisition and analysis of phenotypic data at large scales. This ability to apply more powerful analyses to answer biological questions is a key strength of phenomics approaches more generally. Advances in this area are also being supported by the use of deep learning (Grapov et al., 2018; Nabwire et al., 2021) and non-linear dimensionality reduction (Tills et al., 2021; Tills et al., 2022), both of which can be powerful enablers for dealing with complex systems. Phenomics approaches are now underpinning applications including the move towards personalised medicine, where large datasets of clinical data for individual patients, from molecular to phenotypic levels of biological organisation, are used to inform treatment decisions (Curcin, 2020).

The reasons for phenomics not having been adopted more broadly outside of these areas of research are multifaceted, but likely includes the phenome not being as amenable to transferrable methods as is the case for the subject of molecular omics, combined with a belief that current approaches to phenotyping are adequate. There has been an acknowledged shift (both financial and academic) towards quantifying biological systems at lower levels of biological organisation (Kültz et al., 2013; Noble, 2010), with investment and innovation directed accordingly, towards molecular-omics. Despite the renaissance of whole-organism perspectives on the phenotype in the crop sciences and medicine, it remains the case that integration of phenomics into research areas outside of these disciplines, particularly to non-model species, remains in its infancy.

### Phenomics and Comparative Developmental Physiology

The ability to interrogate an intricate and dynamic biological system is arguably one of the greatest attractions of working with developing organisms, yet it is these same attributes that render their study particularly challenging. Phenomics appears to be a natural complement to this challenge, by enabling the acquisition of phenotypic data at scales that integrate the multifaceted nature of biological responses during this dynamic period of life. Animal development comprises considerable functional and spatial change, with responses to biotic and abiotic factors constituting changes to an array of individual traits. Therefore, using phenomics to study early development brings major advantages, including reducing the element of chance associated with pre-selecting the trait(s) believed to be of functional significance to our biological response of interest. We are not the first to promote a phenomics approach to the study of development–indeed sciomics and high-throughput phenotyping were both proposed in Comparative Developmental Physiology–Contributions, Tools and Trends (Feder, 2006; Spicer, 2006) as being central to the advancement of development, and developmental physiology in particular. Phenomics, however, extends beyond simply widening the dimensionality of phenotypic measurement. Instead, phenomics, and the omics more generally, have the capacity to bring better understanding of biological systems through their analysis in a combinatorial manor, rather than as a sum of their parts. An organism is a functionally integrated unit (Forsman, 2015), and consequently the way in which we measure it should reflect this.

In our own research, we are creating new analytical methods for measuring the phenome of developing organisms. First, as appears to be a frequent precursor to the application of phenomics, we designed and built technologies with which to acquire phenotypic data at whole organism scales, in the form of custom bioimaging hardware–a laboratory instrument for high-throughput and long-term imaging of aquatic embryos (Tills et al., 2018; Tills et al., 2022). The video produced using this approach made immediately clear the limitation of manual approaches to studying a dynamic process. Initially with a focus on developmental events, we were able to observe the complexity surrounding the timing of specific morphological and physiological transitions, such as the onset of cardiovascular function, the initiation of ciliary driven rotation, or more subtle muscular driven behaviours (Tills et al., 2013). Due to the disjunct between the amount of phenotypic information acquired, and what could be feasibly analysed manually by a user, we began considering holistic and non-supervised approaches to measuring development–moving away from trait-specific approaches (Tills et al., 2013; Tills et al., 2022). Most recently, in an attempt to integrate as broad a range of a developing organism’s physiology and behaviour, this has taken the form of an approach termed ‘energy proxy traits’ (Tills et al., 2021). EPTs measure all observable movement-based characteristics of an individual in a video as a spectrum of energy associated with frame-to-frame signals in the brightness of fluctuating mean pixel values. Fluctuations in mean pixel values, the level of brightness in different regions of an image, are the most basic of image statistics. Yet EPTs have proven to be an effective approach to integrating complex developmental physiology (Tills et al., 2022), but also in quantifying cardiac physiology in a range of different species (Ibbini et al., 2022).

**FIGURE 1:**
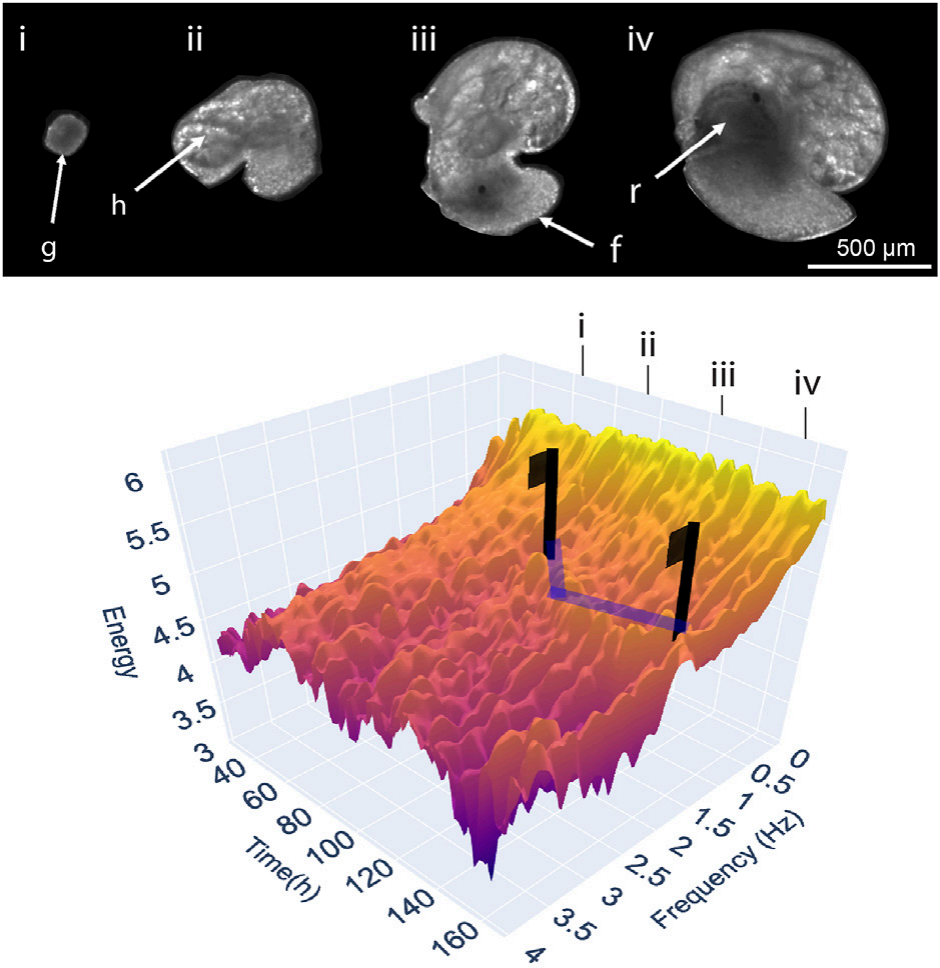
A three-dimensional landscape depicting levels of energy in the EPT spectrum timeseries throughout the embryonic development of an individual freshwater snail *Radix balthica* at (T = 25°C, Tills et al., 2018–Experiment 1). Supplementary Data Sheet S1 contains the data and code necessary for producing the data component of Figure 1, and this can be run online in a Google CoLaboratory Notebook). A multivariate phenotypic response is measured at hourly intervals producing a continuous series, rather than measurement of the timings of discrete points corresponding with the onset of developmental events. Images and annotations indicate the correspondence of key features (g = gastrula, h = heart, f = foot, r = radula) and developmental events (i = the onset of ciliary driven rotation, ii = onset of cardiovascular function, iii = attachment to the egg and onset of muscular crawling, iv = onset of radula function) on the landscape with features in the developing embryo. Blue lines highlight peaks in energy corresponding with frequencies within the range of 1.8–2.5 Hz, associated with the ontogeny of cardiovascular function. Flags are used to illustrate the extent to which discrete measurements miss patterns in the physiological development of the heart, but also the features of the landscape not corresponding to cardiac activity.

Traditional approaches to measuring development can be likened to erecting flags on a high-dimensional landscape (surface), built from a dataset consisting of development (*x*-axis), different phenotypic variables (*y*-axis), and their response (*z*-axis) each depicted along their own axes (Figure 1). Development, and the ontogeny of physiological traits, are frequently treated as a series of discrete events or stages, despite the process itself being a continuum (Burggren, 2021a; b) and their usage largely ignores any phenotypic transition between these periods (‘flags’) in development. This continuous representation of organismal development can be extended, by considering experimental variables such as individual, genotype or experimental treatment as increasingly stacked landscapes, and the differences between them as the net experimental response. Figure 1 portrays such a landscape plotted with real EPT spectra throughout the 7-day embryonic development of the freshwater gastropod *Radix balthica*. The landscape has been annotated indicating the occurrence of major developmental events and transitions, and with vectors indicating the signal associated with the ontogeny of cardiac physiology. Flags placed on the vectors highlighting the signal associated with cardiac physiology indicate how discrete measurement of heart rate at these timepoints would reflect only a small part of the development of heart rate, but also the developmental physiology of the developing embryo more broadly. Within the EPT landscape are troughs linked to manually identifiable transitions in embryo locomotory behaviours, the ontogeny of whole body and gut muscular contractions, but also an integration of all observable organismal physiology much of which is unquantifiable using individual trait-based quantification. Furthermore, considerable variation is also evident at finer scales between these major behavioural and physiological transitions. This stochasticity highlights the implicit variability of movement associated with embryonic behaviours and physiologies on a timepoint-by-timepoint (hourly) basis. We use the flag-landscape analogy to highlight the major limitation of applying limited discrete measurements to the dynamic and continuous system of development. By measuring the phenotype at specific stages in development, considerable changes in observable phenotype are missed (Burggren, 2021a). We use EPTs as they are an approach to development capable of high-dimensional continuous quantification, but a ‘developmental landscape’ could equally incorporate other physiological measures such as growth curves, metabolic rates, or other physiological rates, or indeed be applied to modelling sensitivity (Burggren and Mueller, 2015). Complex developmental landscapes illustrate the power of Comparative Developmental Physiology to contribute to the growth of phenomics as an approach, and to the phenome as a concept.

### What can we learn from where phenomics is established?

Phenomics is most established in research areas where there is a short-term human-benefit to the systematic and high-throughput study of the phenotype, including the selective breeding of crops (Furbank, 2009; Groβkinsky et al., 2015; Neto and Borém, 2015; Lobos et al., 2017; Borrill et al., 2018; York, 2019; Yang et al., 2020; Razzaq et al., 2021) and livestock (Greenwood et al., 2015; Cole et al., 2020; Pérez-Enciso and Stiebel, 2021), biosciences (Čapek et al., 2023) and medicine (Denny et al., 2010; Pendergrass et al., 2011; 2013; Maslov et al., 2018; Özdemir, 2020). Each of these research areas has achieved a technological capacity to study the phenotype with a level of throughput and thoroughness that enables the acquisition of phenome level data; however, subsequent analysis of data at these scales also presents significant challenges. Within the context of animal development, this challenge is magnified given the often considerable functional and morphological changes associated with embryonic and larval development. What then, can we learn from other areas of research that integrate similarly high-dimensional datasets?

Phenomics produces high-dimensional datasets with potentially thousands of predictor-outcome variable relationships (Furbank, 2009; Houle et al., 2010), and its integration into animal development and comparative developmental physiology is thereby limited as much by our ability to analyse and handle data at such scales, as the collection of data themselves (Tardieu et al., 2017). Whilst this is not the place to review the challenges associated with analysing data at these scales, and the statistical techniques used to navigate these challenges, we direct readers to Houle et al. (2010) and Hebbring (2014) for detailed discussion of each of these points. Within the molecular omics, there is fierce and entrenched debate amongst biologists about the potential pitfalls of high-dimensional datasets, and so it seems appropriate to consider these here. Omics datasets can be likened to the mythical Pandora’s box, in that whilst they enable measurement of biology at an unprecedented scale, they also produce vast datasets that can require considerable time and expertise to interpret and can support research pre-hypotheses-testing. Advancement of bioinformatics approaches has undoubtedly been central to the analysis and interpretation of these data, enabling analyses of large omics datasets for both data exploration and hypothesis testing. Integration of phenomics into areas of research including animal development and developmental physiology will therefore necessitate drawing on expertise in bioinformatics and computational biology, all geared towards addressing challenges associated with the acquisition, analysis and storage of data, including handling metadata, ensuring data reproducibility, and driving data standards. As phenomics grows as an approach, and as it is increasingly integrated into a wider diversity of research areas, it becomes increasingly important to learn these lessons from molecular bioinformatics.

### What can Comparative Developmental Physiology contribute to phenomics?

Phenomics as an approach will undoubtedly reward researchers working in developmental biology in their ability to address challenging research questions that capitalise on the breadth and integrated nature of this period of life. However, we also suggest morphological differentiation and physiological ontogeny inherent in organismal development as a model system, has significant potential to develop phenomics as an approach, contributing to advancing many areas of biological research including those identified in Burggren’s (2021a) “Developmental Physiology: Grand Challenges”.

Organismal development is inherently complex, multifaceted and dynamic. Owing to the small size of early life stages and their taxonomic universality, animals during their early development present an excellent model for the application of phenomics, both in a practical sense, and in their capacity to stretch and develop the theoretical understanding surrounding phenomics. Embryos are, after all, used in research ranging from environmental science and ecotoxicology, through to drug discovery and evolutionary developmental biology. The phenome is highly heterogeneous in the types of data required to describe it, and therefore the methods and approaches necessary to measure it. This contrasts markedly with the universality of molecular-omics, with transferrable approaches for both data acquisition and analysis, but also an accessible shared understanding of what each of these molecular-omics are across all areas of biology. Our knowledge of the phenome is crude, and poorly endowed with transferrable approaches compared with the other omics. We therefore support and emphasise the points made by others, that phenomics can act as a key enabler to the advancement of science in the 21st century.

While the potential contribution of embryonic development to advancing the concept of the phenome is multifaceted, we identify four key strengths of developing embryos as models for phenomics:

i) High-dimensional phenotyping of embryos is highly scalable, owing to their small size and ability to be cultured within a laboratory setting. This scalability can enable high-throughput phenotyping approaches of complex phenotypes, with associated large-scale data collection.
ii) Biodiversity, including physiological diversity, mean that application of phenomics to embryonic development not only presents opportunities to advance our understanding of the phenome of this critical period of life across the natural world, but to also accelerate the development of approaches used to measure and analyse it, by drawing on this biodiversity.
iii) Central to phenomics is high-dimensional phenotyping. The information content of the phenome during embryonic development is extremely high (and perhaps greater than at any other life stage), given the considerable temporal, spatial and functional change associated with this period of life. Static trait-based approaches are therefore a well-recognised limitation to its study. Consequently, advancement of technological, theoretical, and analytical approaches to enable the application of phenomics will provide significant opportunity to further our understanding of these dynamic biological systems.
iv) Research to understand the role of embryonic development in evolutionary and ecological processes is broad, and so too are the associated frameworks, models and approaches created to support it. These offer considerable opportunities for the advancement of phenomics, by acting as a scaffold from which to build and interpret new approaches to understanding the phenotype.

### Priorities and future directions

The current pace of technological innovation and change is unparalleled. Consequently the “art of the possible” is rapidly expanding. Undoubtedly this presents significant opportunities to the advancement of phenomics as an approach. However, to capitalise on technological advances it requires a parallel initiative advancing the development of methods, adoption of technologies and training of staff that span both areas. These activities have been central to the advancement of molecular-omics. The pace of technological innovation and the general lack of accessible commercial products in the life sciences has positioned open-source DIY approaches to become common enablers of innovation and research using phenomics. Key strengths of open-source include greater accessibility, accelerated innovation and increased return on investment, but open-source is certainly not a panacea with prerequisite skills, equipment and resources, acting as a potential barrier to adoption. Technological innovation is fuelling the acquisition of phenotypic data at unprecedented scales (Meijering et al., 2016), with bioimaging being one of the greatest contributors to this growth. Here, we have made the case for embryonic development as a model for phenomics, with a focus on how the benefits will be reciprocal. The application of phenomics as an approach, we believe, can lead to significant advancements in our understanding of the evolutionary and ecological significance of changes in the phenotype during early development. In doing so, phenomics as an approach will itself be advanced by using this complex, dynamic and scalable biological system.

The challenge of phenomics is particularly timely given our global biodiversity crisis adding considerable urgency to assessment of biological sensitivity and other conservation physiology themed research activities. Current capacity for phenomics is largely limited to frequently used model species, likely due to the considerable resources associated with developing computational tools capable of measuring high-dimensional phenotypic change (Lussier and Liu, 2007; Xu et al., 2010; Perivali et al., 2018; Olmedo et al., 2015; Lürig et al., 2021). As it stands, phenomics is largely not transferrable taxonomically, regardless of the life stage. As a result, the capacity for phenomics to contribute to research geared towards conservation of biodiversity is currently limited. Comparative Physiology and Comparative Development Physiology operates on the basis of the Krogh principle (Krogh, 1929), in which species are selected based on their suitability to answer the biological question of interest. Consequently, development of high-dimensional phenotyping approaches with levels of transferability comparable to those of the molecular omics should be a priority if we are to facilitate the integration of phenomics into the study of animal development, outside of model species.

In summary, phenomics as an approach provides exciting opportunities for advancing our understanding of the evolutionary and ecological significance of developmental change, the genetic underpinnings of alterations to early development, as well as in interpreting developmental responses to biotic and abiotic change. Directing future research towards the establishment of high-dimensional phenotyping approaches that have the broadest relevance and applicability across the animal kingdom should be a key priority, and here, we identify animal development, particularly developmental physiology, as having significant potential to catalyse this endeavour.

## Data Availability

The original contributions presented in the study are included in the article/Supplementary Material, further inquiries can be directed to the corresponding author.
